# Utilization of Integrated Ayushman Bharat - Pradhan Mantri Jan Arogya Yojana and Mahatma Jyotirao Phule Jan Arogya Yojana at a Tertiary Care Center in Solapur, India: An Epidemiological Study

**DOI:** 10.7759/cureus.74680

**Published:** 2024-11-28

**Authors:** Pravin D Kolekar, Sampatti S Todkar, Sanjay M Mulaje

**Affiliations:** 1 Department of Community Medicine, Dr. V. M. Government Medical College, Solapur, IND

**Keywords:** ayushman bharat, mjpjay, oope, out-of-pocket expenditure, pmjay, public funded health insurance, utilization pattern

## Abstract

Background: The Integrated Ayushman Bharat - Pradhan Mantri Jan Arogya Yojana (AB-PMJAY) and Mahatma Jyotirao Phule Jan Arogya Yojana (MJPJAY) is a public-funded health insurance scheme in the state of Maharashtra. The scheme provides end-to-end cashless services for identified diseases through a network of hospitals from both the government sector as well as the private sector, covered under the scheme. AB-PMJAY is a scheme of the government of India. Such publicly funded health insurance schemes help to reduce out-of-pocket expenditure (OOPE). The study was designed with the objective of describing an epidemiological profile of subjects registered under the Integrated MJPJAY and Pradhan Mantri Jan Arogya Yojana at a tertiary care center and identifying the utilization pattern of the scheme.

Methods: It was a record-based, retrospective cross-sectional study. The study population comprised all the cases registered under Integrated AB-PMJAY and MJPJAY from September 1, 2023, to November 30, 2023, at the tertiary care center. The sample size consists of all the cases (n=1978) registered under the scheme at the tertiary care center from September 1, 2023, to November 30, 2023.

Results: The majority of the subjects (44.39%, n=878) belonged to the age group 40-59 years. In our study, 77.45% (n=1532) of the study subjects got pre-authorization approval. In the case of the pattern of utilization, 56.46% (n=865) of the study subjects utilized medical treatment, and 43.54% (n=667) of the study subjects utilized surgical treatment. In surgical treatment, utilization was higher in polytrauma (15.92%, n=244), followed by general surgery (7.70%, n=118) and ear, nose, and throat (ENT) surgery (5.68%, n=87). In the case of medical treatment, utilization was more in general medicine (14.30%, n=219), followed by nephrology (11.49%, n=176) and neurology (10.64%, n=163).

Conclusion: The majority of the subjects availing themselves of registration in the scheme were in the age group 40-59 years. The majority of the subjects utilized medical treatment. In the case of specialty utilization, polytrauma was utilized more, followed by general medicine. The utilization was least in the case of cardiothoracic surgery, followed by dermatology, hematology, and pediatric surgery.

## Introduction

The World Health Organization defines universal health coverage (UHC) as a means to enable all people and communities to use promotive, preventive, curative, rehabilitative, and palliative health services they need, of sufficient quality to be effective, while also ensuring that the use of such services does not expose the user to financial hardship [1-3]. The International Labour Organization defines health insurance as “the reduction or elimination of the uncertain risk of loss for the individual or household by combining a larger number of similarly exposed individuals or households who are included in a common fund that makes good the loss caused to any one member” [4,5]. A health insurance policy is a contract between an insurance company and an individual and comes in handy in case of severe emergencies [6]. It is an effective social security mechanism [7].

The Government of India launched the Ayushman Bharat - Pradhan Mantri Jan Arogya Yojana (AB-PMJAY) on September 23, 2018. This Yojana is a government-funded health insurance scheme that covers more than 10.74 crore poor and vulnerable families [1]. Mahatma Jyotirao Phule Jan Arogya Yojana (MJPJAY) is a flagship health insurance scheme of the Government of Maharashtra, earlier known as Rajiv Gandhi Jeevandayee Arogya Yojana (RGJAY), was relaunched on April 1, 2017, by the Government of Maharashtra [8,9]. The Integrated AB-PMJAY and MJPJAY have been implemented in revised form across the state from April 1, 2020 [8]. The scheme provides end-to-end cashless services for identified diseases through a network of service providers from the government and private sector [8]. The scheme has many unique features to its credit to proactively reach beneficiaries and guide the beneficiary to avail the services in a cashless manner [9].

The high out-of-pocket expenditure (OOPE) on health care has a devastating effect on the lives of low-income individuals [10]. According to the 75th round (2017-18) of the National Sample Survey, 12% of Indians have unmet health needs [11]. Inability to afford healthcare, i.e., financial inaccessibility, is one of the factors responsible for the unmet needs and, therefore, is a hindrance to UHC in India [11]. OOPE, a major source of health financing in the country, contributes to 49.82% of total health expenditure [12]. In India, in 2018, 16.51% of people faced catastrophic health expenses, i.e., CHE (at a 10% threshold level), and 3.3% of people were pushed into poverty due to OOPE on health [13].

A sizable section of the population may avoid the treatment entirely due to financial constraints. The publicly funded health insurance schemes are helping poor patients by increasing healthcare accessibility and by reducing health expenditure. The study done by Rao et al. about the Rajiv Aarogyasri Community Health Insurance Scheme (RACHIS) in the state of Andhra Pradesh found that it improved access and greater benefits to below the poverty line (BPL) families to secondary and tertiary healthcare [14]. There are many studies done in India about awareness and coverage of publicly funded health insurance schemes across states. There are very few studies done to assess the pattern of utilization of such schemes. In this study, we aimed to know the utilization pattern of this government-funded health insurance scheme at the tertiary care center in Solapur, which has played an important role in the implementation of the scheme.

## Materials and methods

Study design and setting

It was a record-based retrospective cross-sectional study. The study was conducted at a tertiary care center in the Solapur district, India. Permission was obtained from the authorities of Integrated AB-PMJAY and MJPJAY as well as from the authorities of the tertiary care center.

Study population and duration

The study population comprised all the cases registered under the scheme from September 1, 2023, to November 30, 2023, at the tertiary care center. The data of all the registered cases during the three months was collected to ensure consistency and adequate data representation.

Sample size

The sample consisted of all cases (n=1978) registered under the Integrated AB-PMJAY and MJPJAY scheme from the period September 1, 2023, to November 30, 2023, at the tertiary care center.

Inclusion criteria

All the cases registered under the scheme from the period September 1, 2023, to November 30, 2023, at the tertiary care center were included in the study.

Exclusion criteria

No exclusion criteria were applied, as every case registered under the scheme during the study period was included.

Study instrument and data collection

The data for this study was retrospectively collected from the Integrated AB-PMJAY and MJPJAY records of the tertiary care center. All the records of study subjects registered under the scheme during the period from September 1, 2023, to November 30, 2023, were reviewed. The records included socio-demographic information, pre-authorization data, diagnosis, treatment modalities, department, claim status, package, and claim paid amount under the scheme. The information was collected from online records. The required variables were entered in Microsoft Excel. The demographic information of those availing themselves of registration under the scheme, such as age, gender, and district of residence, was collected. The socioeconomic status was determined on the basis of the type of ration card. The pre-authorization data included pre-authorization status approved, rejected, canceled, or pending. Pre-authorization is required for approval to access services under an insurance scheme. Those subjects who got pre-authorization approval from the State Health Assurance Society (SHAS) were eligible for the treatment under the scheme and so could utilize the scheme. The information on diagnosis, type of treatment provided (medical treatment/surgical treatment), and department was collected. The data on claim approval status, package under the scheme, and claim paid amount under the scheme was collected. All the data was anonymized to protect patient confidentiality. Subsequent analysis was carried out.

Statistical analysis

This data was entered in Microsoft Excel and analyzed in Epi Info software (version 7.2). The results were expressed in terms of frequencies and percentages for categorical variables. The analysis was carried out using the chi-square test for goodness of fit for frequency distribution across specialties, and the Z test for proportion was used to check the statistical significance of the proportion of medical treatment.

Ethical considerations

The Institutional Ethics Committee approval was taken from Dr. V. M. Government Medical College, Solapur (approval no.: IEC/169/24). The anonymity and confidentiality of the data were maintained as per ethical guidelines and regulations.

## Results

In our study, the majority of the study subjects (44.39%, n=878) belonged to the age group of 40-59 years. It was followed by 23.15% (n=458) of the study subjects in the age group of 15-39 years and 19.26% (n=381) of subjects in the more than 60 years of age group. The majority of male study subjects as well as female study subjects belonged to the age group of 40-59 years. The number of males was higher in all the age groups except the age group of 40-59 years, where female study subjects were more than males. The findings are outlined in Table 1.

**Table 1:** Age- and sex-wise distribution of study subjects (n=1978)

Age group (years)	Sex				Total	
	Male		Female			
	Frequency (n)	Percentage (%)	Frequency (n)	Percentage (%)	Frequency (n)	Percentage (%)
<1	57	4.81	28	3.53	85	4.30
1-14	109	9.21	67	8.44	176	8.90
15-39	287	24.24	171	21.54	458	23.15
40-59	493	41.64	385	48.49	878	44.39
>60	238	20.10	143	18	381	19.26
Total	1184	100	794	100	1978	100

**Figure 1:**
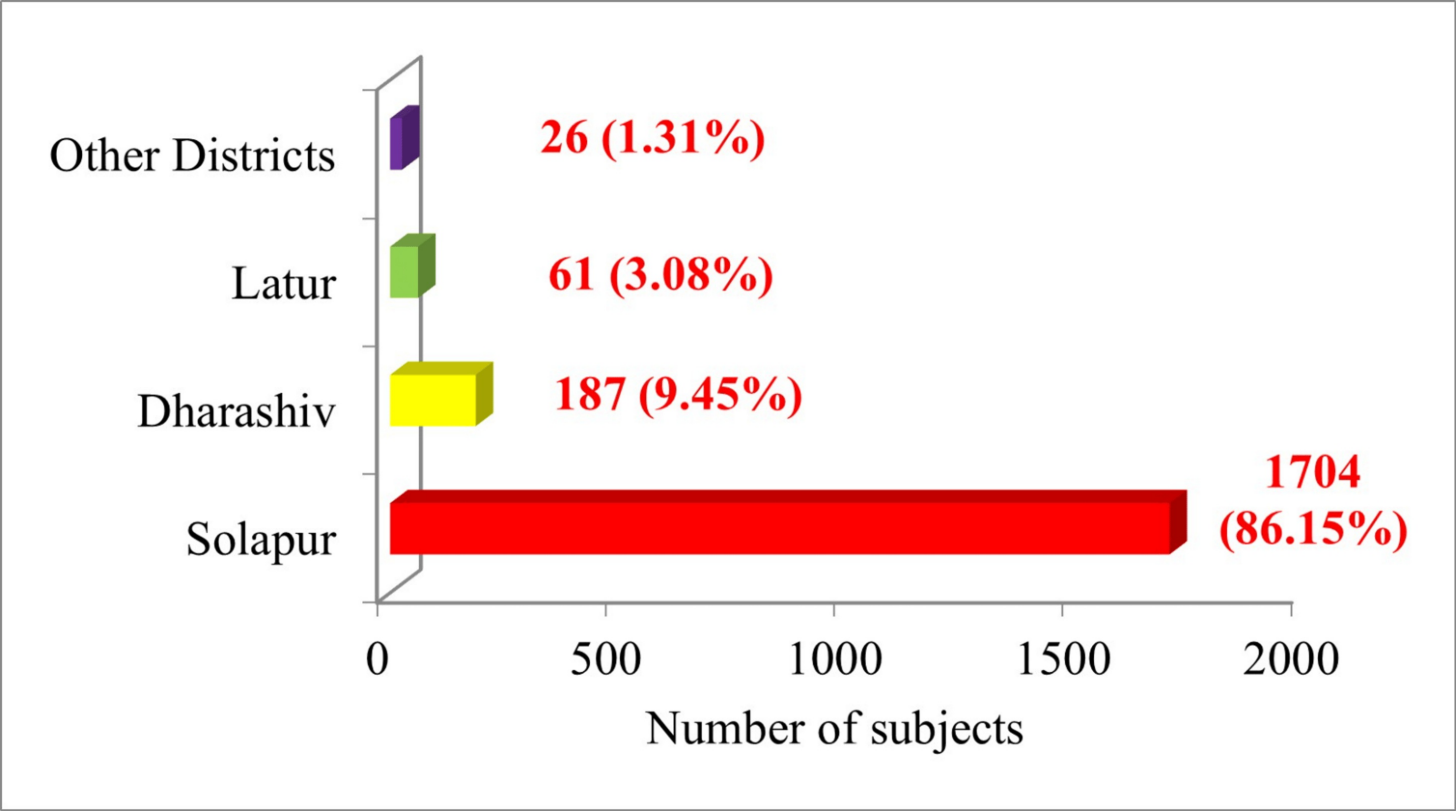
District-wise distribution of study subjects (n=1978)

Most of the study subjects (86.15%, n=1704) were from Solapur district only. It was followed by the Dharashiv district (9.45%, n=187). About 3.08% (n=61) of the study subjects were from the Latur district, and 1.31% (n=26) belonged to other districts. Findings are outlined in Figure 1.

**Figure 2:**
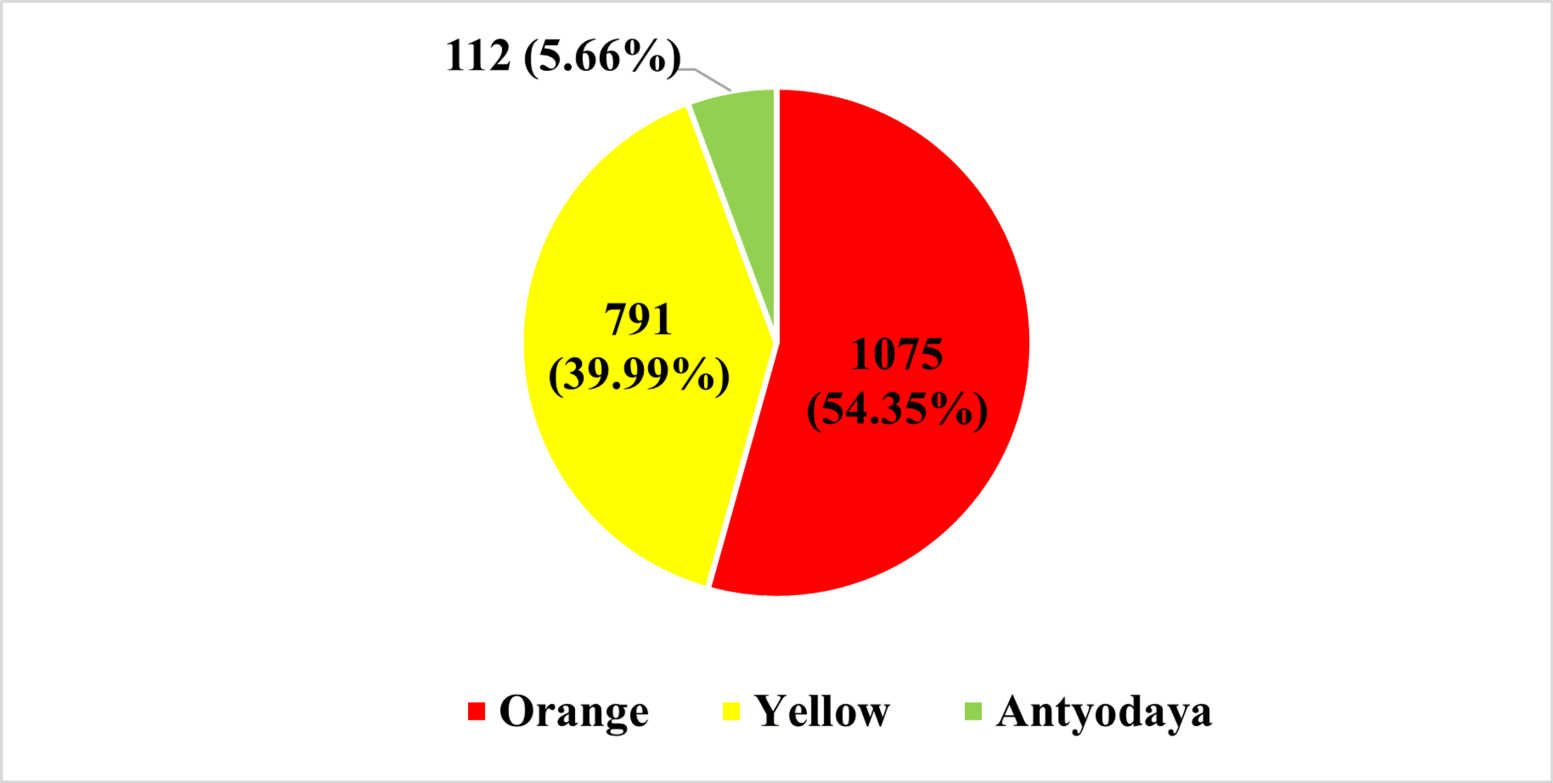
Classification of study subjects on basis of type of ration card (n=1978)

In the study, we found that 54.35% (n=1075) of the study subjects were orange ration card holders. About 39.99% (n=791) were yellow ration card holders, and 5.66% (n=112) of the study subjects held Antyodaya ration cards (Figure 2). A ration card is an official document issued to households as per eligibility to purchase subsidized food grains from the Public Distribution System (PDS).

**Figure 3:**
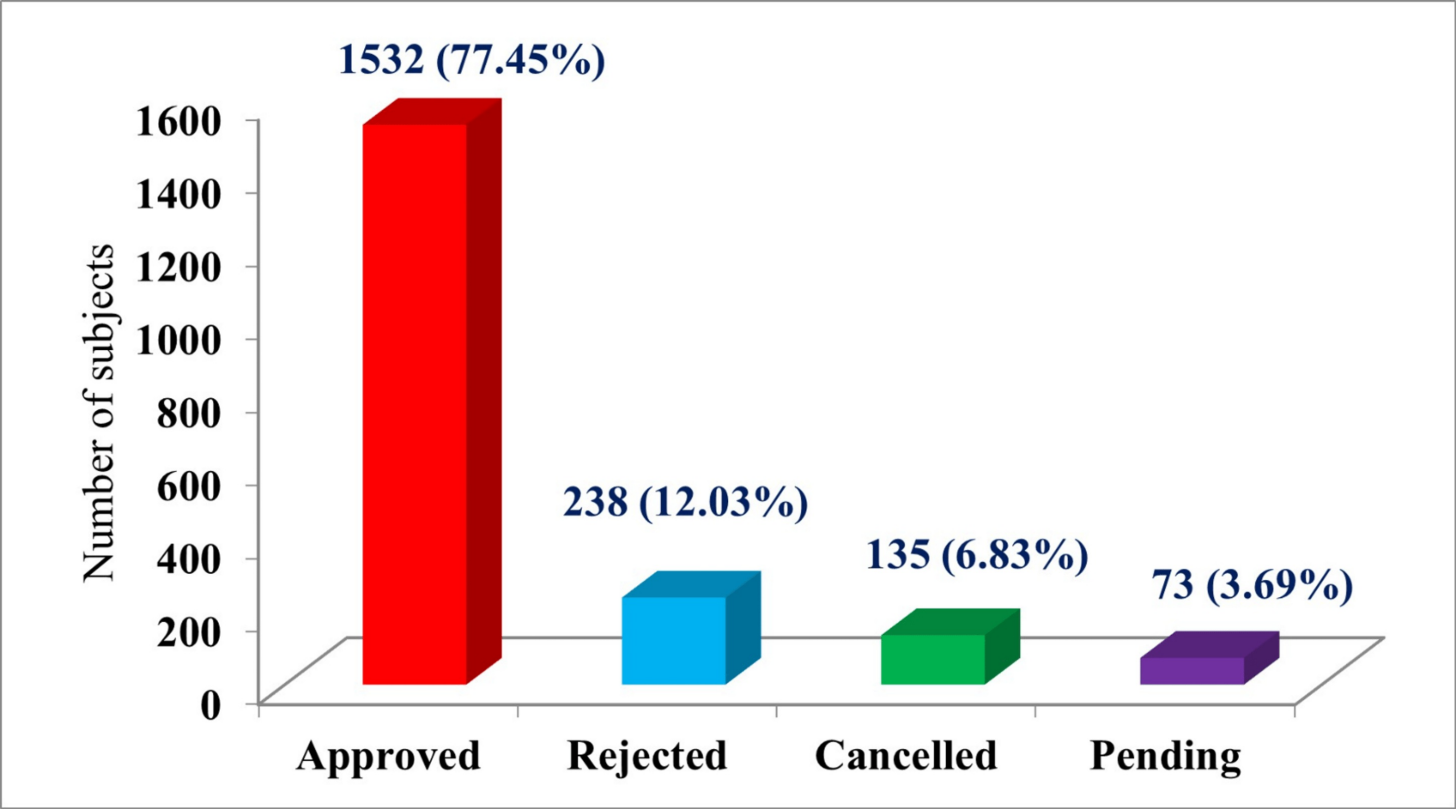
Pre-authorization status of study subjects (n=1978)

In our study, 77.45% (n=1532) of the study subjects got pre-authorization approval from the SHAS. Pre-authorization was rejected in 12.03% (n=238) and was canceled in 6.83% (n=135) of the study subjects. It was pending in the case of 3.69% (n=73) of the study subjects. These findings are highlighted in Figure 3.

**Figure 4:**
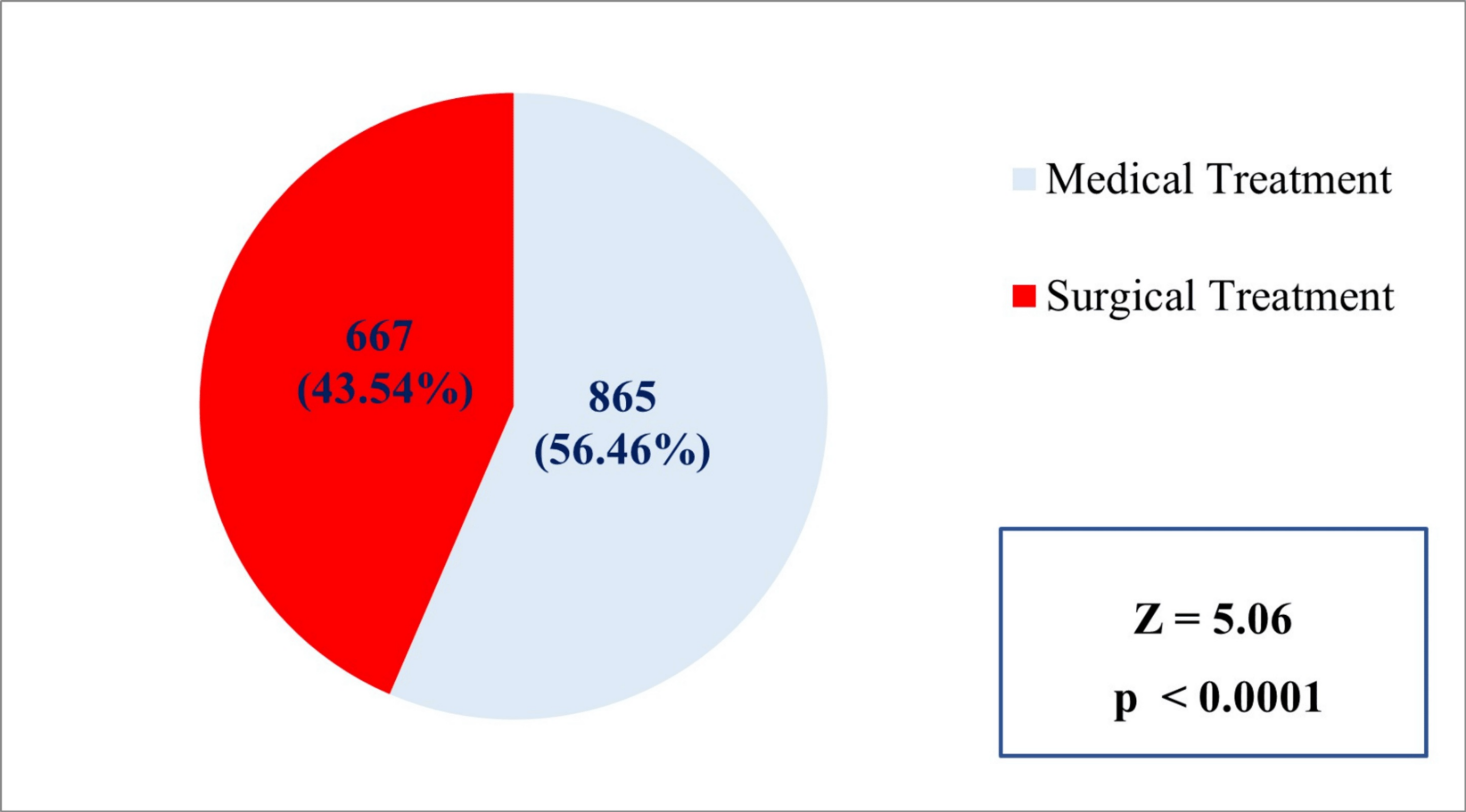
Percentage distribution of patients by type of treatment received (n=1532)* *Number of study subjects who got pre-authorization approval during the study duration and could avail themselves of the treatment under the scheme p=0.00000042 (Z-test for proportion)

In the case of the pattern of utilization, 56.46% (n=865) of the study subjects utilized medical treatment, and 43.54% (n=667) of the study subjects utilized surgical treatment (Figure 4).

Overall, the majority of the study subjects utilized polytrauma (15.92%, n=244). It was followed by general medicine (14.30%, n=219), nephrology (11.49%, n=176), and neurology (10.64%, n=163). In the case of surgical treatment, utilization was more polytrauma (15.92%, n=244). It was followed by general surgery (7.70%, n=118) and ear, nose, and throat (ENT) surgery (5.68%, n=87). In the case of medical treatment, utilization was more in general medicine (14.30%, n=219), followed by nephrology (11.49%, n=176) and neurology (10.64%, n=163). The utilization was least in the case of cardiothoracic surgery (0.07%, n=1), followed by dermatology (0.13%, n=2), hematology (0.13%, n=2), and pediatric surgery (0.32%, n=5). These findings are outlined in Table 2.

**Table 2:** Specialty-wise management of study subjects (n=1532) df: degree of freedom; gynec and obgy: gynecology and obstetrics Chi-square test for goodness of fit was done

Specialty	Subjects		Test
	Frequency (n)	Percentage (%)	
Burns	11	0.72	X²=1794.95, df=22, p<0.001
Cardiothoracic surgery	01	0.07	
Cardiology	23	1.50	
Critical care	11	0.72	
Dermatology	02	0.13	
Endocrinology	22	1.44	
ENT surgery	87	5.68	
Gastroenterology	31	2.02	
General medicine	219	14.30	
General surgery	118	7.70	
Genitourinary system	07	0.46	
Gynec and obgy surgery	39	2.54	
Hematology	02	0.13	
Nephrology	176	11.49	
Neurology	163	10.64	
Neurosurgery	30	1.96	
Orthopedics	72	4.70	
Ophthalmology surgery	31	2.02	
Pediatric surgery	05	0.32	
Pediatrics	122	7.96	
Polytrauma	244	15.92	
Pulmonology	94	6.14	
Surgical oncology	22	1.44	
Total	1532	100	

**Figure 5:**
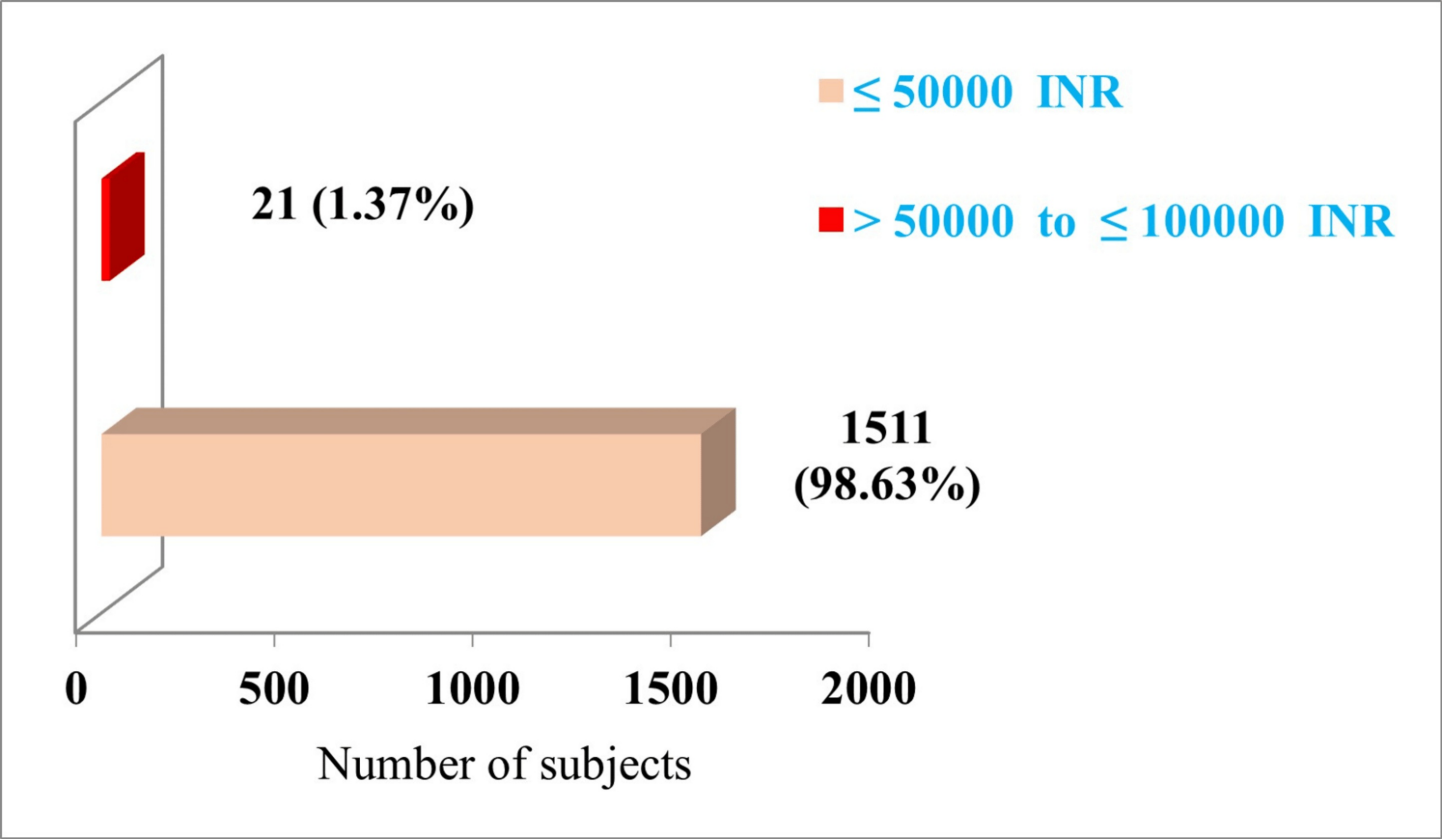
Distribution on basis of claim paid amount (n=1532) INR: Indian Rupees

The claim paid amount in the case of 98.63% (n=1511) of subjects was less than or equal to 50,000 Indian rupees (INR) (≤50,000 INR). In the case of 1.37% (n=21) of subjects, the claim paid amount was greater than 50000 INR to less than or equal to 1,00,000 INR (>50,000 INR to ≤1,00,000 INR) (Figure 5).

## Discussion

In our study, 77.45% (n=1532) of the subjects got pre-authorization approval from SHAS for utilizing the scheme. In the study, the majority of the subjects (56.46%, n=865) utilized medical treatment. Overall, in the case of specialty utilization, more utilization was seen in polytrauma (15.92%, n=244). The utilization was least in the case of cardiothoracic surgery (0.07%, n=1). In the case of 98.6% (n=1511) of the subjects, the claim paid amount was less than or equal to 50,000 INR.

The Niti Aayog report has highlighted non-uniform accessibility to healthcare across the country. There is a disparity between rural and urban India as far as health access is concerned. It is a fact that many families or individuals have to pay out of their own pockets due to unexpected illnesses and a rise in healthcare expenses [15]. Publicly funded health insurance schemes are playing an important role in improving healthcare accessibility and reducing OOPE [16]. Such schemes have been useful in reducing the economic burden of poor and vulnerable families.

In the study, it was found that the majority of the subjects (44.39%, n=878) were aged between 40 and 59 years. The majority of male study subjects as well as female study subjects belonged to the age group of 40-59 years. This age group is the primary beneficiary of this public health initiative. The study done by Radhakrishnan and Bansode-Gokhe had also similar findings [10]. This may be due to the rising burden of non-communicable diseases (NCDs) in this age group. The study done by Naaz and Nigudgi also found that NCD-related claims were maximum, indicating an NCD burden [17]. It was found in the study that the number of males was higher in all the age groups except the age group of 40-59 years, where female study subjects were more than males. This may be due to post-menopausal health problems in the age group along with the burden of NCDs and age-related conditions. This highlights the need for regular health screenings and designing gender-responsive interventions. In the study, the majority of the study subjects (86.15%, n=1704) were from the Solapur district. There is a need as well as opportunities for the decentralization of healthcare services in stepping towards UHC.

We found that the majority of study subjects (54.35%, n=1075) held orange ration cards, representing those above the poverty line. It was followed by yellow ration card holders (39.99%, n=791), representing those BPL. Those BPL may have lower health literacy and lower awareness about the scheme. Similar findings have been noted in a study done by Radhakrishnan and Bansode-Gokhe [10]. It was emphasized by Balarajan et al. that there are differential trends in inequalities suggesting differential uptake and access to healthcare services by different groups [18].

In the study, 77.45% (n=1532) of the study subjects got pre-authorization approval from SHAS. Near similar findings have been reported in the study done by Radhakrishnan and Bansode-Gokhe [10]. Pre-authorization was rejected in 12.03% (n=238) and was canceled in 6.83% (n=135) of the study subjects. It was pending in the case of 3.69% (n=73) of the study subjects. It may be due to inadequate documentation or delay in online updating of data and protocols. The study shows a relatively high pre-authorization approval rate. The rejection and cancellation rates highlight the challenges in providing universal access to healthcare.

In the case of utilization pattern, we found that the majority of the study subjects (56.46%, n=865) utilized medical treatment. About 43.54% (n=667) of the study subjects utilized surgical treatment. In the case of specialty utilization, the majority of the study subjects utilized polytrauma (15.92%, n=244), followed by general medicine (14.30%, n=219).

In the case of surgical treatment, utilization was more in polytrauma (15.92%, n=244), indicating a significant burden of traumatic injuries. It was followed by general surgery (7.70%, n=118) and ENT surgery (5.68%, n=87). In the case of medical treatment, utilization was more in general medicine (14.30%, n=219), consistent with the rising burden of chronic NCDs. It was followed by nephrology (11.49%, n=176) and neurology (10.64%, n=163), indicating the demand for healthcare services for acute and chronic conditions related to kidneys and the nervous system through publicly funded health insurance schemes. The utilization was least in the case of cardiothoracic surgery (0.07%, n=1), followed by dermatology (0.13%, n=2), hematology (0.13%, n=2), and pediatric surgery (0.32%, n=5). The study done by Naaz and Nigudgi found that overall medicine department claims (PMJAY) were at 42% [17].

The claim paid amount in the case of the majority of study subjects (98.6%, n=1511) was less than or equal to 50,000 INR. These findings emphasize further capacity building of tertiary care hospitals and frequent package revisions. 

It is reported in the study by Rao et al. that inpatient care is only consumed by a small proportion of households in a year [19]. The lack of adequate knowledge among the beneficiaries is the concern for not utilizing such schemes. The study done by Thomas et al. in three districts of Gujrat found that 43.3% of beneficiaries utilized the benefit of the Ayushman Bharat scheme [20]. The study done by Sriee and Maiya among 300 households in rural areas found that 47.24% of the households availed themselves of the Ayushman Bharat scheme [15].

It is recommended based on the findings of the study that the training of resident doctors and/or doctors and health care workers in the empaneled hospitals needs to be done at regular intervals so as to facilitate an increase in the pre-authorization approval and availing of the scheme. There is a need to improve the communication between healthcare workers and beneficiaries. The awareness at the level of empaneled hospitals can be increased through information, education, and communication (IEC) and through a community needs assessment approach. Media platforms will help to increase the outreach of such schemes. The local television channels and radio will be helpful in broad community outreach. Social media platforms like Facebook, X (Twitter), and Instagram can help in targeted interactive communication. These platforms can help to deliver concise information about the scheme. Those who have successfully availed themselves of the scheme can become the champions of the scheme to deliver the message to the community. This will help increase awareness as well as encourage the community to utilize the scheme. There is an urgent necessity to adopt comprehensive preventive approaches. The policy intervention UHC at the level of government will help increase healthcare access and reduce OOPE.

## Conclusions

The study highlights that utilization was least in specialties such as cardiothoracic surgery, dermatology, hematology, and pediatric surgery. There is a need for increasing awareness of such specialties along with strengthening super specialty services as well as, at the level of policy-making, having a comprehensive review of packages in such specialties. The study findings also highlight the scope for further capacity building in trauma care and chronic disease management. The study, through the understanding of patterns of utilization, will help policy stakeholders with resource allocation, healthcare policy, and future research initiatives.
